# Adipocytes as a Link Between Gut Microbiota-Derived Flagellin and Hepatocyte Fat Accumulation

**DOI:** 10.1371/journal.pone.0152786

**Published:** 2016-04-01

**Authors:** Eveliina Munukka, Petri Wiklund, Tiina Partanen, Sakari Välimäki, Eija K. Laakkonen, Maarit Lehti, Pamela Fischer-Posovzsky, Martin Wabitsch, Sulin Cheng, Pentti Huovinen, Satu Pekkala

**Affiliations:** 1 Department of Health Sciences, University of Jyväskylä, Jyväskylä, Finland; 2 Department of Clinical Microbiology and Immunology, University of Turku, Turku, Finland; 3 LIKES Research Center for Sport and Health Sciences, Jyväskylä, Finland; 4 Division of Pediatric Endocrinology and Diabetes, University Medical Center Ulm, Ulm, Germany; 5 Department of Physical Education, Shanghai Jiao Tong University, Shanghai, China; College of Tropical Agriculture and Human Resources, University of Hawaii, UNITED STATES

## Abstract

While the role of both elevated levels of circulating bacterial cell wall components and adipose tissue in hepatic fat accumulation has been recognized, it has not been considered that the bacterial components-recognizing adipose tissue receptors contribute to the hepatic fat content. In this study we found that the expression of adipose tissue bacterial flagellin (FLG)-recognizing *Toll-like receptor* (TLR) *5* associated with liver fat content (r = 0.699, p = 0.003) and insulin sensitivity (r = -0.529, p = 0.016) in humans (n = 23). No such associations were found for lipopolysaccharides (LPS)-recognizing *TLR4*. To study the underlying molecular mechanisms of these associations, human HepG2 hepatoma cells were exposed *in vitro* to the conditioned culture media derived from FLG or LPS-challenged human adipocytes. The adipocyte-mediated effects were also compared to the effects of direct HepG2 exposure to FLG and LPS. We found that the media derived from FLG-treated adipocytes stimulated fat accumulation in HepG2 cells, whereas either media derived from LPS-treated adipocytes or direct FLG or LPS exposure did not. This is likely due to that FLG-treatment of adipocytes increased lipolysis and secretion of glycerol, which is known to serve a substrate for triglyceride synthesis in hepatocytes. Similarly, only FLG-media significantly decreased insulin signaling-related Akt phosphorylation, *IRS1* expression and mitochondrial respiratory chain ATP5A. In conclusion, our results suggest that the FLG-induced *TLR5* activation in adipocytes increases glycerol secretion from adipocytes and decreases insulin signaling and mitochondrial functions, and increases fat accumulation in hepatocytes. These mechanisms could, at least partly, explain the adipose tissue *TLR5* expression associated with liver fat content in humans.

## Introduction

The prevalence of non-alcoholic fatty liver disease (NAFLD) is increasing, currently affecting 14–24% of the general population and up to 80% of morbidly obese individuals [1]. The pathophysiology of NAFLD is complex and has not been fully elucidated. However, growing evidence indicates that inflammation and insulin resistance are important factors in its causation. Previous studies suggest that the inflammatory processes in NAFLD are caused by mitochondrial dysfunction, oxidative stress and subsequent lipid peroxidation [2]. While the metabolic consequences of NAFLD are well documented, the exact underlying mechanisms are not well understood. Recently, the bacterial surface molecules originating from gut microbiota in hepatic fat accumulation has been acknowledged [3,4]. Animal studies have shown that altered gut microbiota composition can cause intestinal inflammation, leading to leakage of gut bacterial components or translocation of entire commensal bacteria to the circulation and peripheral tissues of the host [5–7]. Our recent findings in humans suggest that adipose tissue inflammation could be the key link between the gut microbiota-derived bacterial molecules and NAFLD [3]. Specifically, subjects with high hepatic fat content overexpressed inflammatory *Vav1*, *Lat* and *MMP-9* in their subcutaneous adipose tissue, which, in addition to function in leukocyte transendothelial migration, are components of the Toll-like receptor (TLR) signaling pathway.

TLR family members are key mediators of the innate immune system. They recognize bacterial surface molecules, such as lipopolysaccharides (LPS) from the outer wall of Gram-negative bacteria and flagellin (FLG), which is a structural protein of flagellum, a bacterial locomotive organelle. TLR activation leads to the pleiotropic activation of various inflammatory and metabolic genes [8]. Previous studies have shown that three TLR family members (TLR2, 4, and 9) play a role in the development of NAFLD [9]. For the FLG-recognizing TLR5, contradictory roles have been reported [10–12]. Of the pattern recognition receptors of the innate immune system that sense the exogenic pathogens also Nod-like receptors (NLRs) have been recognized as links between immune function and metabolism [13]. By far, pyrin domain containing 3 (NLRP3) inflammasome is the most studied inflammasome of the NLR family. NRLP3 expression has been found to be increased in the adipose tissue of subjects with metabolic syndrome and insulin resistance [14], and more recently its role in regulating NAFLD progression has been recognized [10].

Our recent results showed that in adipocytes bacterial FLG and LPS increased secretion of glycerol [15], which serves as a substrate for hepatic triglyceride synthesis. In insulin resistant adipose tissue glycerol is produced by increased lipolysis. In this study, therefore, we hypothesized that adipose tissue expression levels of TLR4 and TLR5, and possibly other TLRs and NOD-like receptors would associate with liver fat content and insulin resistance in humans. In order to verify the associations and establish the adipocytes as a link between gut-derived molecules and hepatocytes, cell cultures were selected as a model instead of animals to eliminate any confounding factors.

## Materials and Methods

### Human Subjects

Participants were recruited from a larger study (the AMB-study The Finnish Academy SKID-KID program), conducted at the University of Jyväskylä in accordance with the Helsinki Declaration and approved by the ethical committee of the Central Finland Health Care district. A written informed consent was obtained from all study participants. To exclude the confounding effect of sex, only women were included in this study. All together 23 women (age 18–57 years old) were included. The health history and the current status were checked by a study physician. There was no type 1 or 2 diabetes, cardiac diseases, autoimmune diseases or major liver (cancer, hepatitis) diseases. All subjects were clinically euthyreotic.

The subjects underwent a 75-g oral glucose tolerance test (OGTT). Whole-body insulin sensitivity was calculated from glucose and insulin values during the OGTT as proposed by Matsuda and DeFronzo [16]. Glucose was measured using KONELAB 20XTi analyzer (Thermo Fischer Scientific inc.) and serum insulin was determined by immunoassay using the IMMULITE Analyser (Diagnostic Products Corporation, Los Angeles).

The subcutaneous adipose tissue biopsies were taken under sterile conditions between 7 and 9 am after overnight fasting. Biopsies were frozen in liquid nitrogen and stored at -80°C until used. Total RNA was isolated using FastPrep systems (MP Biomedicals, France) and RNeasy Lipid Tissue Mini Kit (Qiagen, Valencia, CA, USA), amplified and processed using the GeneChip 3´IVT Express Kit (Affymetrix, Santa Clara, CA, USA) and hybridized on Affymetrix Human Genome U219 Array Plates as described previously [3]. From the microarray data adipose tissue Toll-like receptors, NOD-like receptors and several lipolysis-associated gene expression levels were determined. The array data of this study have been submitted to ArrayExpress (E-MTAB-2649).

The assessment of hepatic fat content was performed by ^1^H MRS (1.5T GE Signa CV/i scanner, GE Medical Systems, Waukesha, WI, USA) as described in Munukka et al. 2014.

### Cells Lines, Cultures and Reagents

SGBS preadipocytes [17] were maintained in DMEM/F12 supplemented with 10% FBS (Invitrogen), 0.17 μM panthotenat plus 0.33 μM biotin (P/B, Sigma Aldrich) and penicillin/streptomycin solution (Invitrogen). Preadipocytes were differentiated into mature adipocytes by incubating them first for 4 days in DMEM/F12 supplemented with P/B, 0.01 mg/ml transferrin, 20 nM insulin, 100 nM cortisol, 0.2 nM triiodothyronine, 25 nM dexamethasone, 250 μM IBMX and 2 μM rosiglitazone (QUICK DIF MEDIUM) (all from Sigma-Aldrich), and afterwards in DMEM/F12 supplemented with P/B, 0.01 mg/ml transferrin, 20 nM insulin, 100 nM cortisol and 0.2 nM triiodothyronine (3FC MEDIUM) for 10 days. When SGBS adipocytes were exposed to FLG for RNA extraction, the 14-days differentiated adipocytes in 3FC MEDIUM were treated for 1 and 4 hours with 10 ng/ml of FLG.

When HepG2 cells were exposed to the conditioned SGBS culture media, 14-days differentiated adipocytes in 3FC MEDIUM were treated for 24 hours with 100 ng/ml of LPS or 10 ng/ml of FLG. These concentrations were determined to efficiently affect adipocytes in our former study ^13^. Afterwards the culture media was collected and centrifuged for 5 min at 300xg. The freshly collected conditioned culture media was then applied to HepG2 cells that were previously washed twice with PBS.

HepG2 hepatoma cells (from ATCC) were maintained in DMEM/Glutamax media supplemented with 10% FBS, 100 U/mL penicillin, 100 μg/mL streptomycin, and 1% sodium pyruvate (all from Invitrogen, Carlsbad, CA, USA). When HepG2 cells were exposed to recombinant *Salmonella typhimurium* FLG (Invivogen, San Diego, CA, USA) and *Escherichia coli* LPS (Sigma-Aldrich, St Louis, USA), FBS was omitted from the media and the cells were previously washed twice with PBS (Invitrogen). To mimic the conditions used for adipocytes, for the direct HepG2 exposures LPS was used at a concentration of 100 ng/ml and FLG at 10 ng/ml.

### Immunofluorescence

SGBS adipocytes were grown and allowed to differentiate for 14-days on cover slips. To study the effects of FLG treatment on lipid droplets, FLG at 10ng/ml was added to the culture media for 3 hours. Afterwards the cells were washed twice with PBS and fixed for 15 min with 4% PFA-PBS, permeabilized with 0.5% Triton-X for 5 min, blocked 1 hour with 5% donkey serum and thereafter incubated with primary antibody over night at 4°C. Immunolabeling was performed using rabbit polyclonal antibody against TLR5 (Pierce, Appleton, WI, USA, 1:50 in 1% donkey serum) and mouse monoclonal antibody against perilipin (Progen, Heidelberg, Germany, 1:200 in 1% donkey serum). As secondary antibodies donkey anti-mouse Alexa Fluor 555 and donkey anti-rabbit 488 (Invitrogen) were used. The labeled cells were imaged using an inverted wide-field microscope (Carl Zeiss) with a confocal unit and 40× oil/1.4 NA objective (Carl Zeiss). To count the percentage of cells in which lipid droplets were degraded, 200 control and FLG-treated cells were observed in randomly selected fields using 63× oil/1.4 NA objective.

### RNA Extraction and Real-Time Quantitative PCR

HepG2 and SGBS cells were homogenized in Trizol reagent (Invitrogen) and the total RNA was extracted according to the supplier’s protocol. Total RNA was reversely transcribed according to the manufacturer’s instructions using High Capacity cDNA Synthesis Kit (Applied Biosystems, Foster City, CA, USA). Real-time PCR analysis was performed according to MIQE guidelines using in-house designed primers (from Invitrogen), iQ SYBR Supermix and CFX96™ Real-time PCR Detection System (Bio-Rad Laboratories, Richmond, CA, USA). The primer sequences were as follows: *IRS1*: Fwd5’ TATGCCAGCATCAGTTTCCA’3 and Rev5’ GGATTTGCTGAGGTCATTTAGG’3; *DGAT2*: Fwd5’ GAGGGGTCTGGGAGATGG’3 and Rev 5’ CCTGTAGCTGCTTTTCCACC’3; *β-actin*: Fwd5’ AGAGCTAGCTGCCTGAC’3 and Rev5’GGATGCCACAGGACTCCA’3; *MMP9*: Fwd5’GAGTGGCAGGGGGAAGATGC’3 and Rev 5’CCTCAGGGCACTGCAGGATG’3; *NFκB* (p65): Fwd5’ATGGCTTCTATGAGGCTGAG’3 and Rev5’CACAGCATTCAGGTCGTAGT’3; *GAPDH*: Fwd5’CCACCCATGGCAAATTCC’3 and Rev5’TGGGATTTCCATTGATGACAA’3; *ATGL*: Fwd5’CAACGCCACGCACATCTA’3 and Rev5’ACCTCAATGAACTTGGCACC’3; *MGL*: Fwd5’TATGAAGAGCTGGCTCGGAT’3 and Rev5’TACCATCCTCTCCCCTTCG’3; *FABP3*: Fwd5’GCACCTGGAAGCTAGTGGAC’3 and Rev5’GTGGTAGGCTTGGTCATGCT’3, and *PLIN1*: Fwd5’ACCTTGCTGGATGGAGACC’3 and Rev5’AGGTCTTCTGGAAGCATTCG’3.

All results from HepG2 cells were normalized to the expression of *β-actin* and from the SGBS cells to the expression of *GAPDH*. The relative expression levels for each gene were calculated with the ΔΔ*C*_*t*_ method (by setting a fold change value of 1 to the average Ct value of the control samples). The amplification efficiency for each gene was 100±2%.

### Protein Extraction and Western Blot

Proteins from HepG2 cells were extracted at 4°C using an ice-cold lysis buffer (10 mM Tris-HCl, 150 mM NaCl_2_, 2 mM EDTA, 1% Triton X-100, 10% glycerol and 1 mM DTT), supplemented with protease and phosphatase inhibitors (Sigma Aldrich). 20–30 micrograms of protein extracts were separated by SDS-Page using 4–20% Criterion gradient gels (Bio-Rad Laboratories, Richmond, CA) and transferred to nitrocellulose membranes. After blocking, the membranes were probed overnight at 4°C with primary antibodies purchased from Cell Signaling Technology (Danvers, MA, USA) (p-Akt), Sigma-Aldrich (anti-GAPDH) and Abcam (MitoProfile® Total OXPHOS Rodent WB Antibody Cocktail, Abcam, Cambridge, MA, USA). Odyssey anti-rabbit IRDye 800CW and anti-mouse IRDye 680RD (LI-COR Biosciences, Lincoln, NE, USA) were used as secondary antibodies. Finally, the blots were scanned and quantified by using Odyssey CLX Infrared Imager of Li-COR and manufacturer's software. If re-probing was needed, the membranes were incubated for 10 min in 0.2 M NaOH at RT, washed with TBS and re-probed with appropriate antibodies. All samples and results were normalized to GAPDH.

### Oil Red O Staining for Neutral Lipid Measurement in HepG2 Cells

Oil Red O stain (Sigma-Aldrich) was used to stain the neutral triglycerides and lipids in HepG2 cells. Briefly, the HepG2 cells on 12-well culture plates were exposed for 24 hours either directly to FLG and LPS, or to the culture media derived from treated adipocytes. Afterwards, the cells were fixed with 10% formalin, and then washed with H_2_O and 60% isopropanol. After washing, the cells were incubated with Oil Red O staining solution at RT for 10 min. Before eluting the staining solution with 100% isopropanol and measuring the absorbance at 550 nm, the cells were washed several times with H_2_O.

### ROS Measurement from HepG2 Cells by Luminescence

For ROS measurement the HepG2 cells were treated with FLG or LPS or with the culture media from treated adipocytes for 24 hours. ROS were detected using non-lytic ROS-Glo™ H2O2 Assay and Glomax Multidetection System (Promega, Madison, WI, USA) according to the manufacturer’s instructions.

### Glycerol Measurement from the Adipocyte Cell Culture Media

SBGS adipocytes were allowed to differentiate for 14 days. For glycerol measurement the cells were treated with FLG for 3 hours. Afterwards the cell culture media was collected and centrifuged for 5 min at 300xg. Glycerol was measured using the KONELAB 20XTi analyzer (Thermo Fischer Scientific inc.).

### Statistical Analysis

All data were checked for normality using the Shapiro-Wilk´s test in PASW 18.0 for Windows. HepG2 cell culture data were analyzed using ANOVA. Bonferroni post hoc tests were performed to localize the effects for the results. Data obtained from FLG-treated SGBS cells were analyzed using Kruskal-Wallis. Spearman correlation analyses were performed to determine the relationship between adipose tissue gene expression and clinical characteristics. The level of significance was set at p<0.05.

## Results

### Correlation of Adipose Tissue *TLR4*, *TLR5* as well as Other TLRs and NOD-Like Receptors mRNA with Liver Fat Content and Insulin Sensitivity in Humans

**Fig 1:**
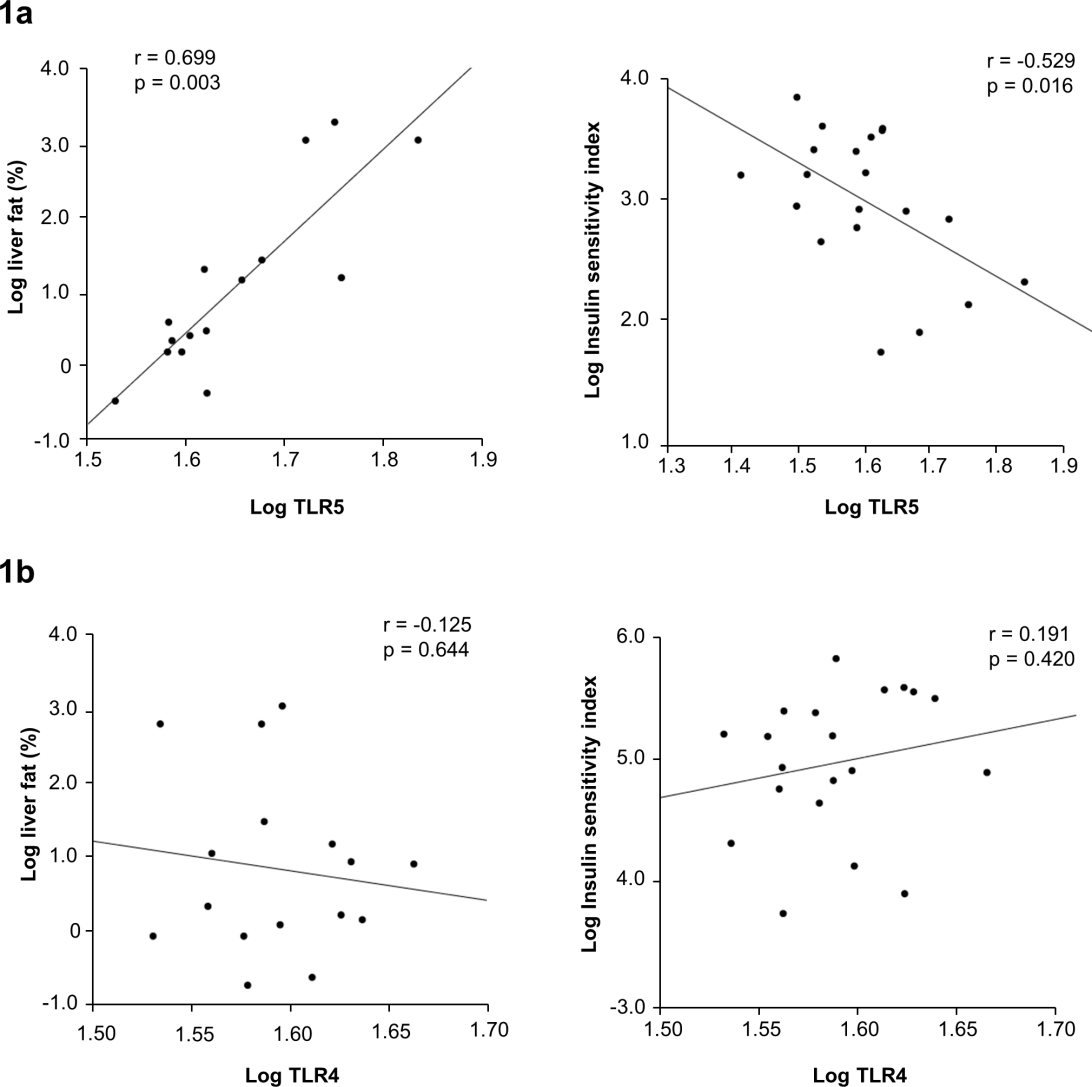
Correlation of liver fat content and Matsuda index (= insulin sensitivity) with (A) adipose tissue *TLR5* mRNA and (B) adipose tissue *TLR4* mRNA in humans (n = 23).

The mean age of the study subjects was 34 years ± 15 years. All subjects were normoglycemic and three subjects had non-alcoholic fatty liver based on the clinical diagnostic cut-off criterion (liver fat content > 5.6%). Adipose tissue *TLR5* expression correlated closely with liver fat content (r = 0.699, p = 0.003) and insulin sensitivity index (r = -0.529, p = 0.016, Fig 1A). *TLR4* expression was not associated with liver fat content or insulin sensitivity (Fig 1B). Of the other TLRs and NOD-like receptors *TLR1* (r = -0.71, p<0.001), *TLR2* (r = -0.599, p = 0.005), *TLR7* (r = -0.687, p = 0.001), *TLR8* (r = -0.641, p = 0.002), *NLRC4* (r = -0.505, p = 0.023) and *NLRP3* (r = -0.628, p = 0.003) associated negatively with insulin sensitivity index. In addition to *TLR5* only *NLRP3* correlated positively with liver fat content (r = 0.545, p = 0.029).

### The Effects of FLG and LPS on Lipid Metabolism and Lipid Content of the HepG2 Cells

**Fig 2:**
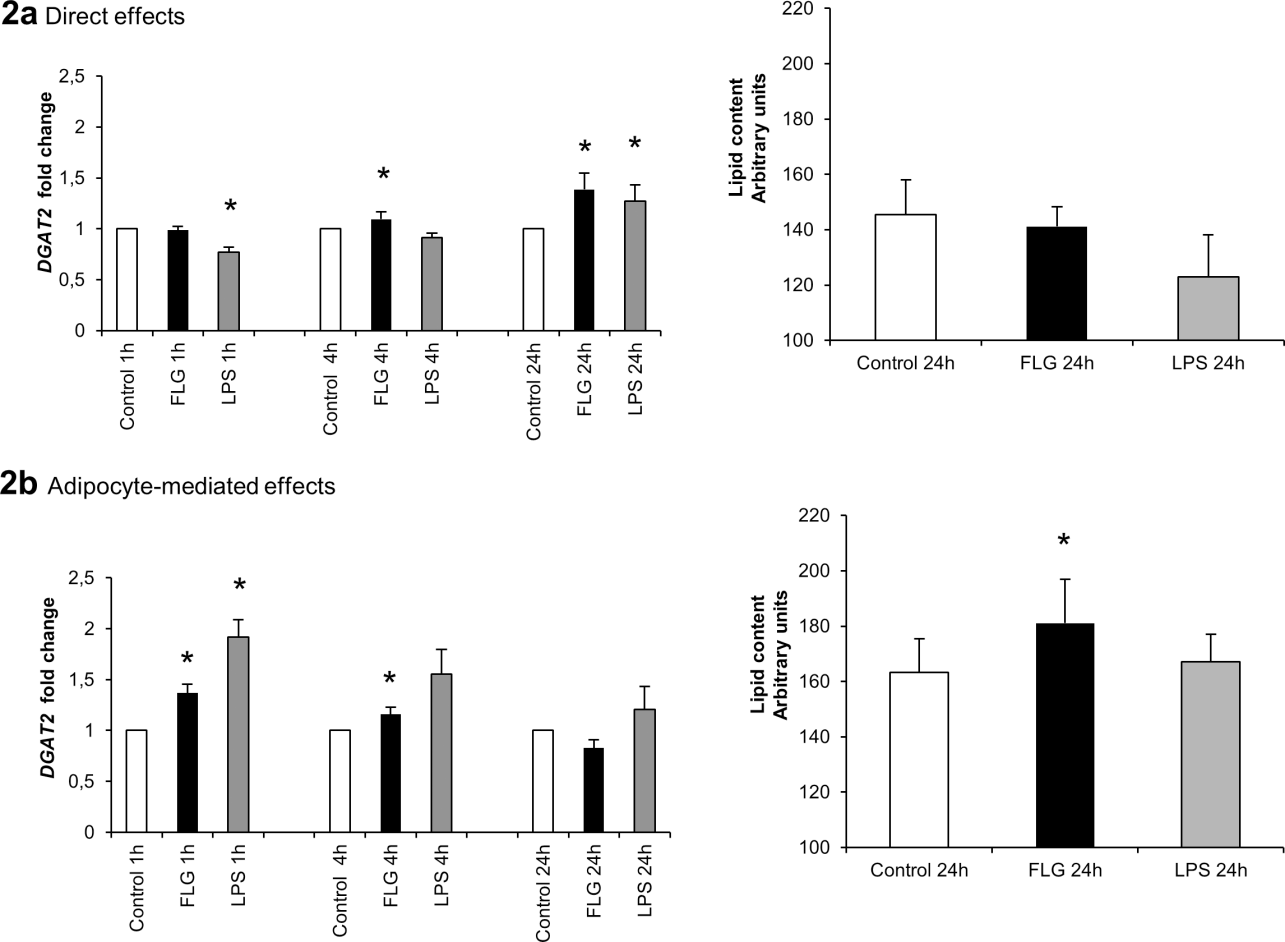
**The (A) direct and (B) adipocyte-mediated effects of flagellin and LPS on the lipid metabolism of HepG2 cells.** The figure presents the direct and adipocyte-mediated effects of FLG and LPS on the *DGAT2* mRNA fold changes. The values are means + SD (n = 5). The results were normalized to *β-actin* and the fold changes were calculated with the *ΔΔC*_*t*_ method. The bars on the right side present graphically the total lipid content of HepG2 cells measured spectrophotometrically using Oil Red O after (A) direct 24 hours exposure to FLG and LPS, and (B) 24 hours exposure to conditioned media derived from FLG and LPS-treated adipocytes. The values are means + SD (n = 12). * indicates a p-value of <0.05.

We further determined the underlying mechanisms linking FLG to hepatic fat accumulation and explaining the lack of association between *TLR4* and hepatic fat content. For the purpose, HepG2 cells were either exposed directly to FLG and LPS or to the conditioned culture media derived from FLG- and LPS-challenged adipocytes. The direct exposure of hepatoma cells to LPS increased triglyceride-synthesizing Diglyceride acyltransferase 2, *DGAT2* expression after 24 hours, and to FLG after 4 and 24 hours (p<0.05). However, the direct FLG or LPS exposure did not increase HepG2 intracellular lipid content (Fig 2A).

Exposure of HepG2 cells to the conditioned culture media from FLG and LPS-treated adipocytes increased *DGAT2* expression at 1 hour, and in addition FLG-treated conditioned media increased *DGAT2* expression at 4 hours (p<0.05 for all). The lipid content of the hepatoma cells was moderately increased only by the exposure to the media from FLG-treated adipocytes (p<0.05, Fig 2B).

### FLG Exposure Causes Glycerol Secretion from Adipocyte Lipid Droplets by Increasing Expression of Lipolytic Genes

**Fig 3:**
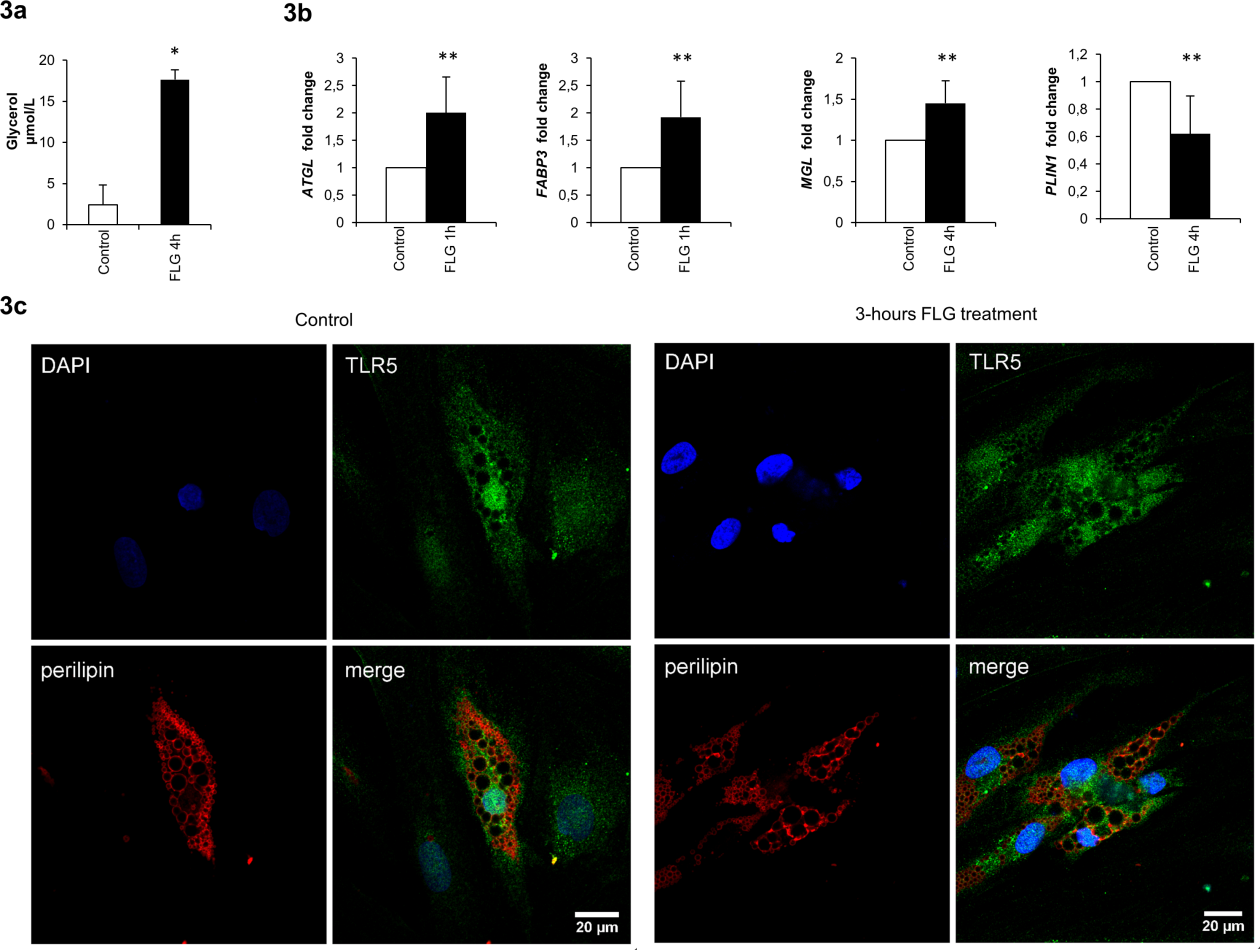
Glycerol is secreted from adipocyte lipid droplets due to increased lipolysis in response to flagellin treatment. (A) Adipocytes were treated with FLG for 3 hours and glycerol was measured from the cell culture media. The values are means + SD (n = 4). * indicates a p-value of <0.05. (B) Adipocytes were treated with FLG for 1 and 4 hours and changes in the expression of *ATGL*, *PLIN1*, *FABP3* and *MGL* mRNA were determined. The values are means + SD (n = 4). ** indicates a p-value of <0.001. The results were normalized to *GAPDH* and the fold changes were calculated with the *ΔΔC*_*t*_ method. (C) Adipocytes with and without 3-hours FLG treatment were labeled with TLR5 and perilipin antibodies, and imaged with confocal microscope. Compared to 30% in control cells, in 70% of FLG-treated adipocytes degradation of lipid droplets membranes was observed (perilipin-labeling in red). In mature adipocytes TLR5 is mainly located in intracellular pool near lipid droplets and nuclei without co-localization, and only a minority on cell membranes.

The exposure of adipocytes to FLG increased glycerol secretion into the culture media (p<0.05, Fig 3A). We further determined the effects of FLG treatment on lipolysis-related gene expression to reveal the mechanisms behind glycerol release. We found that after 1 hour exposure to FLG the adipocytes expressed more adipose triglyceride lipase (ATGL) and fatty acid binding protein 3 (FABP3), and after 4 hours more monoglyceride lipase (MGL) and less perilipin-1 (PLIN1) (p<0.001 for all, Fig 3B). Accordingly, in human adipose tissue *TLR5* correlated positively with *FABP3* (R = 0.549, p = 0.007) and negatively with *PLIN1* (R = -0.631, p = 0.002).

To visualize whether the secreted glycerol was derived from adipocyte lipid droplets, FLG-treated adipocytes were labeled with antibody against lipid droplet membrane protein, perilipin. After 3 hours FLG induced lipid droplet degradation, observed as disintegration of lipid droplet membranes (Fig 3C). Lipid droplet degradation was observed in 70% of FLG-treated adipocytes, and only in 30% of untreated control cells. In addition, we confirmed our earlier findings of that only a minority of TLR5 pool located on cell membranes and the intracellular pool near nuclei and lipid droplets without any co-localization with those structures [15].

### The Effects of FLG and LPS on Inflammation in HepG2 Cells

**Fig 4:**
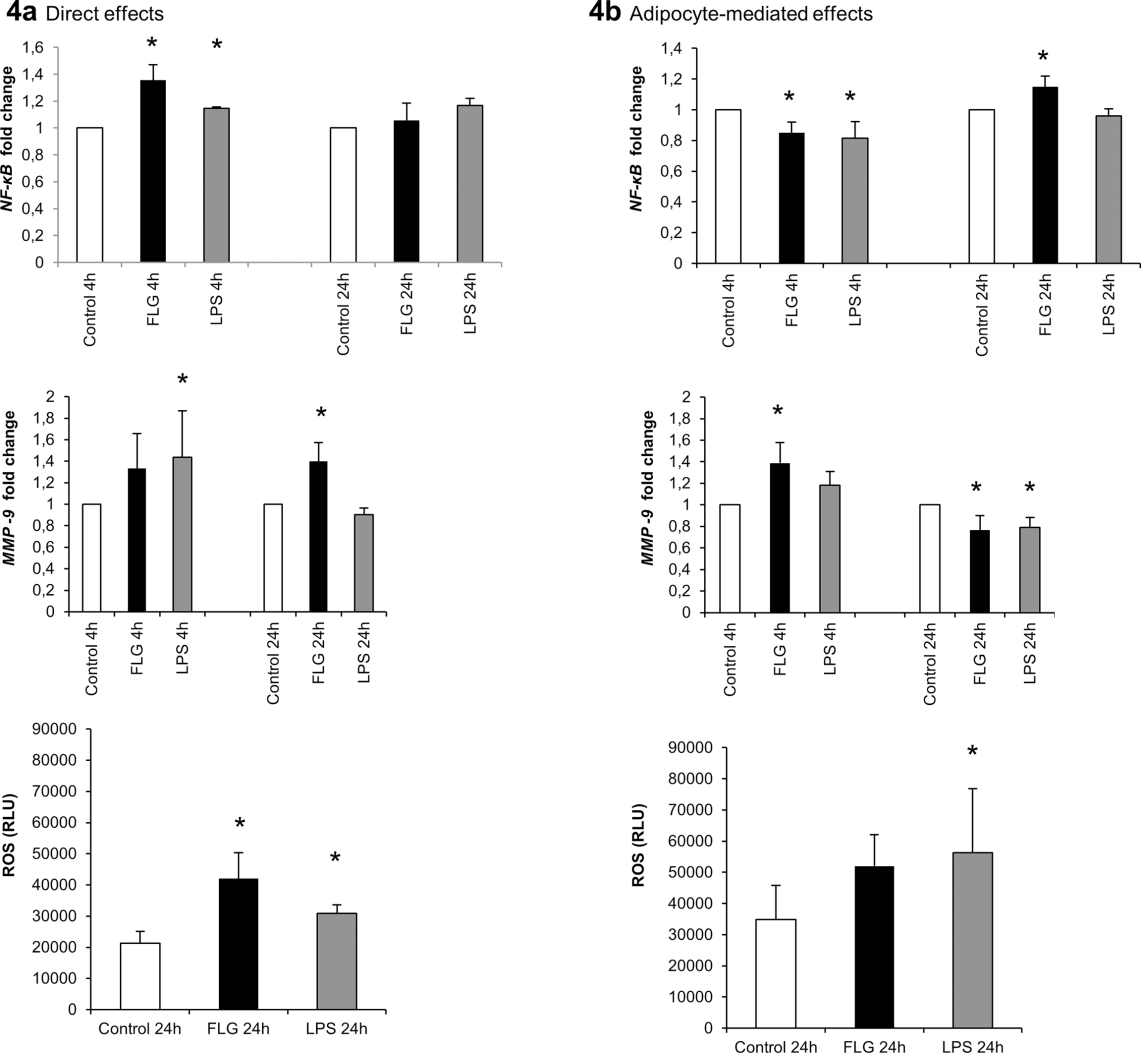
**(A) The direct, and (B) adipocyte-mediated effects of FLG and LPS on inflammation in HepG2 cells.** The figure presents the mRNA fold changes of *NF-κB* and *MMP-9* in response to 4 and 24 hour treatments. The results were normalized to *β-actin* and the fold changes were calculated with the *ΔΔC*_*t*_ method. The values are means + SD (n = 5). The bars below present graphically the amount of luminometrically measured ROS produced after 24 hours exposure. The values are means + SD (n = 5). * indicates a p-value of <0.05.

After the direct exposure of HepG2 cells, both FLG and LPS increased cytokine production-related Nuclear factor kappa B, *NF-κB* (p65) expression after 4 hours (p>0.05 for both, Fig 4A). In addition, LPS increased extracellular matrix-degrading Matrix metalloprotease-9, *MMP-9* mRNA at 4 hours and FLG at 24 hours (p<0.05 for both).

Contrary to the direct exposures, the conditioned media from both the FLG and LPS-treated adipocytes decreased *NF-κB* (p65) expression slightly at 4 hours, and FLG-media increased it at 24 hours (p<0.05 for all, Fig 4B). *MMP-9* mRNA levels were increased at 4 hours and then decreased after 24 hours exposure (p<0.05 for all).

The inflammatory reactive oxygen species (ROS) production increased after exposing the HepG2 cells directly to FLG and LPS (p<0.05 for both, Fig 4A). In addition, the conditioned media from LPS-treated adipocytes increased ROS production, and FLG tended to increase it (p = 0.06, Fig 4B).

### The Effects of FLG and LPS on Insulin Signaling in HepG2 Cells

**Fig 5:**
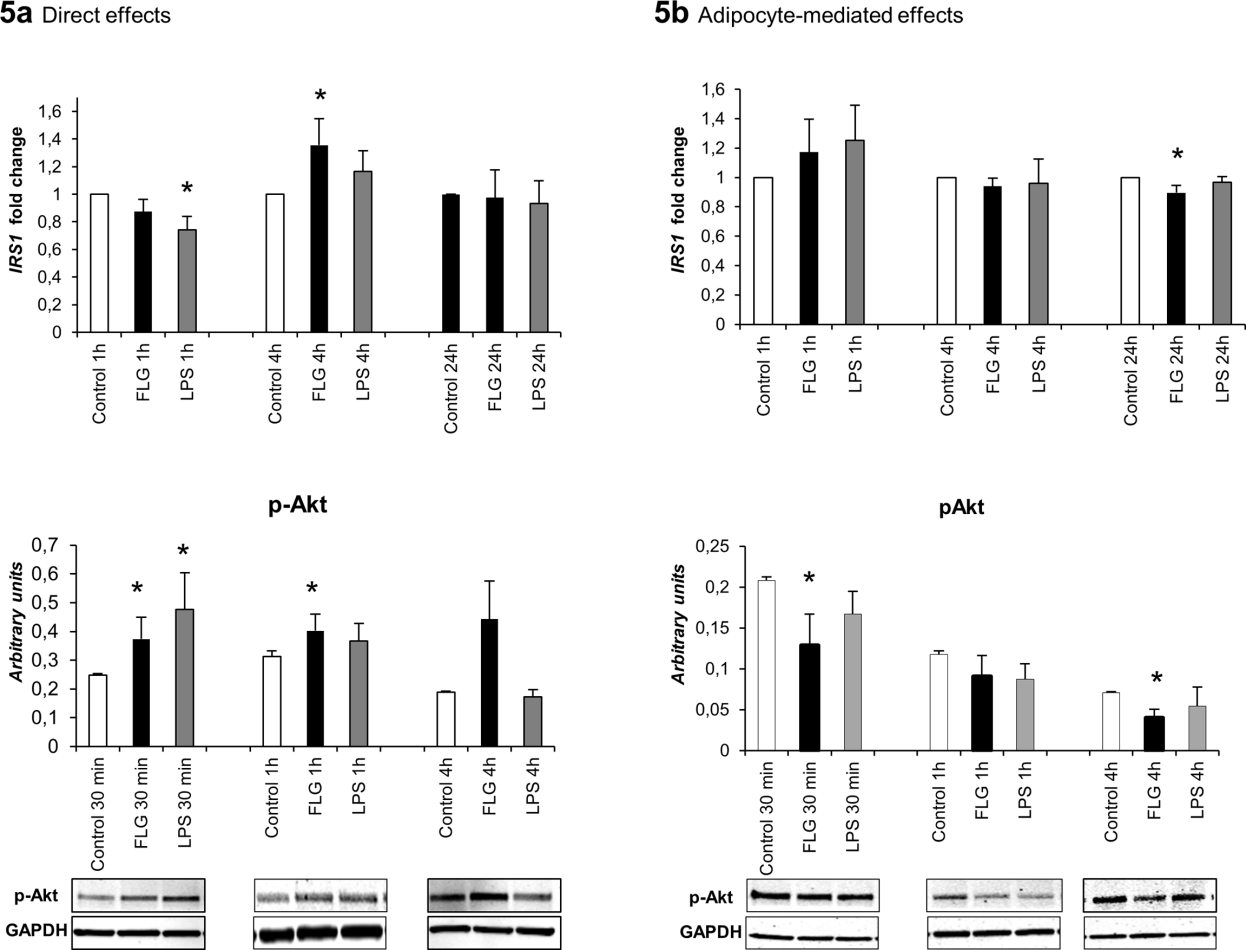
**(A) The direct, and (B) adipocyte-mediated effects of FLG and LPS on insulin signaling in HepG2 cells.** The figure presents the mRNA fold changes of *IRS1* in response to 1, 4 and 24 hour treatments (n = 5) and the phosphorylation levels of Akt in response to 30 min, 1 and 4 hour treatments (n = 4). The mRNA results were normalized to *β-actin* and the fold changes were calculated with the *ΔΔC*_*t*_ method. Akt phosphorylation levels were normalized to GAPDH and the determined intensities of the Western blot bands are presented as arbitrary units. The values are means + SD. * indicates a p-value of <0.05.

The direct exposure of hepatoma cells to LPS moderately decreased insulin receptor substrate 1 (*IRS1*) expression at 1 hour, whereas FLG increased *IRS1* expression at 4 hours (p<0.05 for both, Fig 5A). Phosphorylation of Akt downstream in insulin signaling pathway increased in response to both LPS and FLG treatments (p<0.05).

The conditioned media from FLG-treated adipocytes decreased *IRS1* mRNA at 24 hours slightly but significantly (p<0.05, Fig 5B), and by almost 2-fold the phosphorylation of Akt after 30 min and 4 hours exposure (p<0.05 for both).

### The Effects of FLG and LPS on the Expression of Mitochondrial Respiratory Chain Complex Subunits in HepG2 Cells

**Fig 6:**
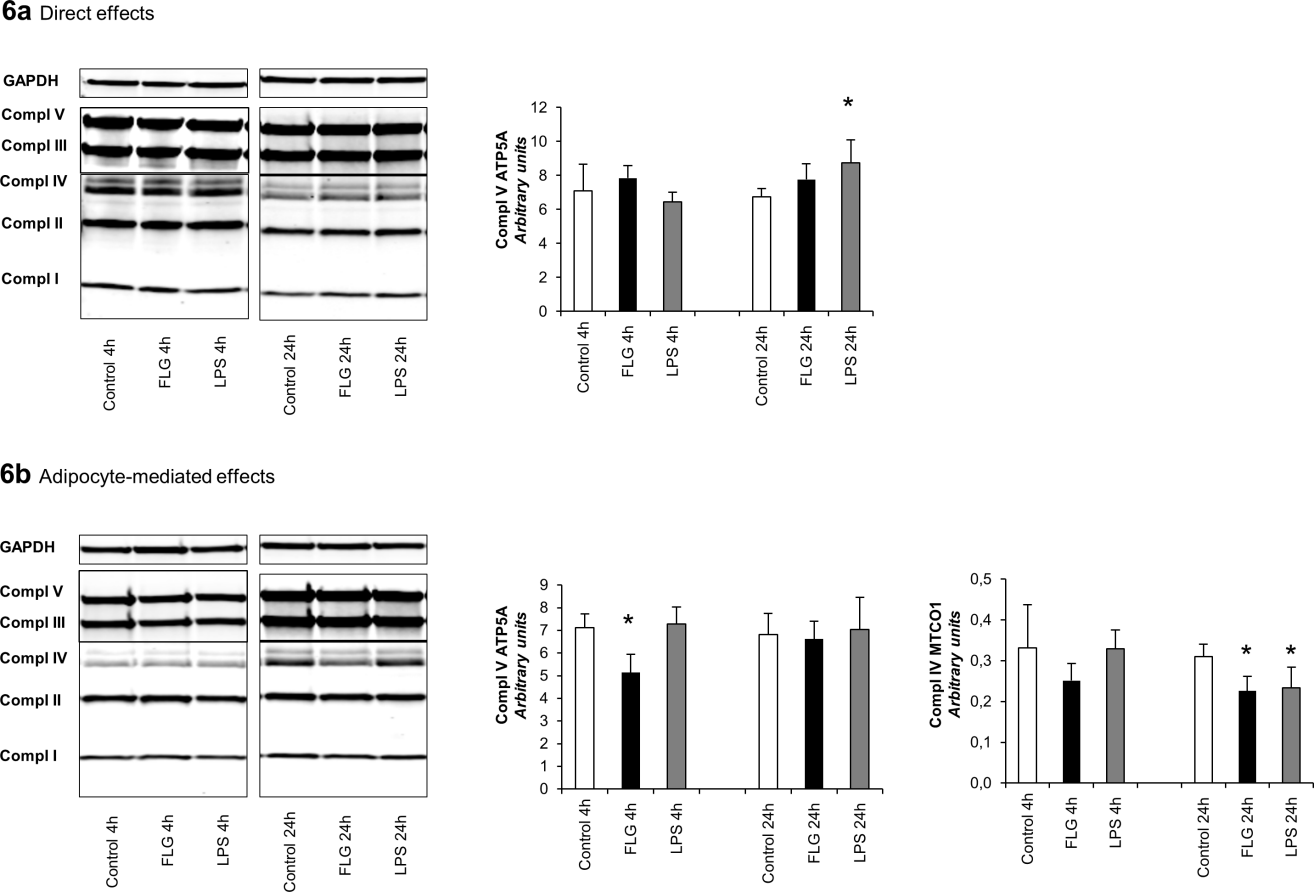
**(A) The direct and (B) adipocyte-mediated effects of FLG and LPS on the expression of mitochondrial respiratory chain complex subunits in HepG2 cells.** The figure presents the expression levels of mitochondrial respiratory chain complex subunits ATP5A (Compl V), UQCRC2 (Compl III), MTCO1 (Compl IV), SDHB (Compl II) and NDUFS8 (Compl I) in response to 4 and 24 hours exposure to FLG and LPS. All expression levels were normalized to GAPDH and the determined intensities of the Western blot bands are presented as arbitrary units. The values are means + SD (n = 3). * indicates a p-value of <0.05.

The direct exposure of hepatoma cells to FLG and LPS did not exert major effects on the mitochondrial respiratory chain (MRC) complex subunits from I to V, except that LPS treatment increased ATP synthase subunit ATP5A protein expression (p<0.05, Fig 6A). Contrarily, conditioned media derived from FLG-treated adipocytes decreased ATP5A protein expression, and both FLG and LPS media decreased Cytochrome C oxidase subunit MTCO1 expression (p<0.05 for all, Fig 6A).

## Discussion

The gut-liver axis is currently considered an important pathway in the development and progression of NAFLD but the underlying mechanisms remain largely unknown. Elevated levels of circulating bacterial surface molecules, such as LPS have been reported in patients with NAFLD and in animal models [18–21]. In addition, the role of adipose tissue in liver fat accumulation has been recognized [22]. However, the possibility that gut bacterial molecules contribute to the onset of NAFLD through adipose tissue has not been considered, even though the gut microbiota is recognized as a plentiful source of for instance bacterial FLG-compartments that further induce various physiological and immunological processes in host. In this study, we found a close correlation of liver fat content and insulin sensitivity with FLG-recognizing adipose tissue *TLR5* expression levels but not with LPS-recognizing *TLR4* that has been previously linked to NAFLD. Our *in vitro* studies also suggest that exposure of human hepatoma cells to the conditioned culture media of FLG-treated adipocytes but not LPS increase triglyceride synthesis and moderately the lipid content in the cells. The underlying mechanisms in HepG2 cells observed in this study involve adipocyte-derived glycerol as well as insulin signaling and mitochondrial functions.

The results showing that FLG-recognizing adipose tissue *TLR5* contributes to the liver fat accumulation *in vivo* are in agreement with our previous hypothesis that adipose tissue inflammation acts as a link between gut microbiota (or surface molecules derived from them) and NAFLD [3]. Interestingly, no associations between *TLR4* and liver fat content were found, which is consistent with our previous findings of that human subjects with high liver fat content did not differ on plasma LPS levels from subjects with low liver fat content [3]. However, these results are different from several others showing an important role for TLR4 in NAFLD [9]. It may be that the effects of TLR4 *in vivo* are not universal and depend largely on the gut microbiota composition, gut integrity and/or the bacterial species of which the circulating LPSs are derived.

Of the other TLRs peptidoglycan-recognizing *TLR1* and *TLR2*, and single stranded RNA-recognizing *TLR7* and *TLR8* associated with insulin sensitivity, which is in agreement with their recognized role in linking inflammation to metabolism [23]. Of the NOD-like receptors *NRLC4* that has been shown to be the cytosolic receptor for flagellin [24] and *NRLP3* also associated with insulin sensitivity. However, besides *TLR5* only *NRLP3* correlated with liver fat content. *NRLP3* has been previously linked to NAFLD [10], obesity and insulin resistance [25]. It is thought to be activated in response to various stimuli (e.g. ssRNA and LPS) including entire pathogenic agents [26]. Nevertheless, it remains unclear what the real ligand for NLRP3 is. Studies have shown that it is activated by ROS suggesting that it would happen upon TLR-induced ROS production [26]. This theory would be in agreement with our previous study showing that exposure of human adipocytes to FLG increased *TLR5* expression and ROS production [15].

Our results show that FLG increases glycerol secretion from adipocytes through increased lipolysis and lipid droplet degradation. Decreased insulin actions in adipocytes are known to enhance lipolysis [27] and as we have previously shown that FLG decreases insulin signaling [15] it is a plausible link between FLG and lipolysis. In the lipolytic pathway down-regulation of PLIN1 up-regulates ATGL [27], which can be observed also in our results. ATGL breaks the triglycerides down to monoglycerides and MGL monoglycerides to glycerol, which transport from adipocytes is facilitated by FABP3 [28]. These pivotal steps of lipolysis in adipocytes were affected by FLG exposure indicating that FLG regulates lipolysis.

The glycerol released from the adipocytes is expected to increase triglyceride synthesis in hepatocytes as in liver, *de novo* fat synthesis pathway enzymes use carbohydrates and glycerol derived from adipose tissue lipolysis as substrates. Glycerol produced by the adipose tissue lipolysis *in vivo* is taken up by the liver and used for lipid synthesis [29]. Interestingly, despite that the direct exposures also induced *DGAT2* expression, fat accumulated in hepatoma cells only when the cells were challenged with media derived from FLG-treated adipocytes. This may, at least partly, be due to that while the media derived from FLG-treated adipocytes decreased Akt phosphorylation and thus insulin signaling, the direct exposures of HepG2 cells increased it. The increased Akt phosphorylation in HepG2 cells in response to the bacterial surface molecules could be due to the negative feedback mechanism for TLR-driven inflammation [30]. Nevertheless, altogether the normal mitochondrial respiration and insulin signaling can retain the correct balance between fat synthesis, oxidation and clearance of triglycerides thus preventing the hepatoma cells from accumulating fat, when the cells are directly exposed to bacterial surface molecules. Notwithstanding the exact underlying regulatory mechanisms warrant further attention.

We further studied the underlying mechanisms that might be involved in hepatocyte fat accumulation in response to FLG. Our results show that culture media from FLG-treated adipocytes, which contains glycerol, decreased the expression of MRC complex subunits, and tended to increase ROS production. Decreased MRC and ATP synthase activity have been reported in NAFLD patients [31]. When fat accumulates in hepatocytes and the impaired MRC is coupled to increased fatty acid oxidation, ROS are increasingly produced and instigate the cellular metabolism and inflammatory reactions [32]. Thus, it seems that both inflammation and uptake of glycerol contribute to fat accumulation in HepG2 cells, while the direct exposure of the cells to FLG only increases ROS production maintaining normal intracellular lipid content. Our findings are in agreement with studies showing that TLRs and lipid accumulation by itself increase ROS production and inflammation [33,34]. Dysfunctional MRCs have been also linked to decreased insulin signaling in liver [2], which is in agreement with the results of this study.

During the progression of NAFLD inflammatory lesions finally result in fibrosis. Hepatic fibrogenesis is preceded by the initial degradation of extracellular matrix permitting infiltration of inflammatory cells to the liver and described finally by the deposition of extracellular matrix. One of the factors regulating the fibrogenic process are ROS that, among others, modify the expression of matrix metalloproteases (MMP) [35]. In our study, FLG and LPS through their effects on adipocytes first increased and then decreased *MMP-9* expression, which would be expected when extracellular matrix is first degraded and then deposited. The direct exposure of HepG2 cells acutely and in longer term resulted in an increase of *MMP-9* expression indicating that the fibrogenic processes may not be activated. However, our findings are in agreement with previous studies showing that TLRs regulate MMP expression [36] and profibrogenic responses [37], and that lipid accumulation itself activates inflammatory responses and profibrogenic gene expression in hepatocytes [38].

The weakness of this study is that adipocytes were not separated from other adipose tissue-resident inflammatory in human biopsies. Therefore, we cannot exclude that for instance macrophages may also have contributed to *TLR5* expression levels in humans. However, this setback does not exclude the importance of the relatively moderate but clear effects of FLG-treated adipocytes on hepatoma cells *in* vitro suggesting that adipocyte-hepatocyte axis constitutes one pathway that leads to hepatocyte fat accumulation. In addition, the cell cultures findings are limited by the fact we did not vary insulin concentrations. Further studies are needed to determine whether insulin boosts or hinders the effects of FLG and LPS on hepatocytes.

We conclude that adipose tissue FLG-recognizing *TLR5* and not LPS-recognizing *TLR4* associates with liver fat content and insulin sensitivity in humans. The *in vitro* studies revealed the underlying mechanisms by showing that the TLR5 activation in adipocytes induced by flagellin may enhance hepatic fat accumulation by decreasing insulin signaling and mitochondrial functions and increasing triglyceride synthesis due to increased lipolysis and glycerol secretion from adipocytes.

## Abbreviations

FLG: flagellin
NAFLD: non-alcoholic fatty liver disease
LPS: lipolysaccharides
TLR: Toll-like receptor
DGAT: Diglyceride acyltransferase
ROS: reactive oxygen species
NF-κB: Nuclear factor kappa B
MMP-9: Matrix metalloprotease 9
IRS1: Insulin receptor substrate 1
MRC: mitochondrial respiratory chain
AS160: TBC1 domain family member 4, Akt substrate of 160 kDa
ATP5A: ATP synthase, H+ transporting, mitochondrial F1 complex, alpha subunit
UQCRC2: Cytochrome b-c1 complex subunit 2
MTCO1: Mitochondrially encoded cytochrome c oxidase I
SDHB: Succinate dehydrogenase complex, subunit B, iron sulfur (Ip)
NDUFS8: NADH dehydrogenase (ubiquinone) Fe-S protein 8.
