# Correction: Adipocytes as a Link Between Gut Microbiota-Derived Flagellin and Hepatocyte Fat Accumulation

**DOI:** 10.1371/journal.pone.0157254

**Published:** 2016-06-03

**Authors:** Eveliina Munukka, Petri Wiklund, Tiina Partanen, Sakari Välimäki, Eija K. Laakkonen, Maarit Lehti, Pamela Fischer-Posovszky, Martin Wabitsch, Sulin Cheng, Pentti Huovinen, Satu Pekkala

The seventh author’s name is spelled incorrectly. The correct name is: Pamela Fischer-Posovszky
